# Management of distal choroidal artery aneurysms in patients with moyamoya disease: report of three cases and review of the literature

**DOI:** 10.1186/1477-7819-11-187

**Published:** 2013-08-12

**Authors:** Kangmin He, Wei Zhu, Liang Chen, Ying Mao

**Affiliations:** 1 Department of Neurosurgery, Huashan Hospital, Fudan University, Shanghai 200040, China

**Keywords:** Aneurysm, Intracranial hemorrhage, Revascularization, Moyamoya disease

## Abstract

Prevention of rebleeding plays an important role in the treatment of hemorrhagic moyamoya disease, because rebleeding results in high mortality and morbidity. We discuss possible treatment for patients with moyamoya disease accompanied with distal choroidal artery aneurysms and review the literature to summarize clinical treatment and mechanisms. The cases of three male patients who suffered from intraventricular hemorrhage are presented. Computed tomography (CT) and digital subtractive angiography (DSA) revealed that bleeding was believed to be caused by ruptured aneurysms originating from distal choroidal artery aneurysms. Two patients successfully underwent superficial temporal artery (STA)-middle cerebral artery (MCA) bypass combined with encephalo-duro-myo-synangiosis (EDMS) and the obliteration of the aneurysm. The follow-up DSA or CT scan demonstrated that the aneurysms completely disappeared with the patency of the reconstructed artery. Neither of the patients experienced rebleeding during the follow-up period (up to 34 months). Given conservative treatment, the third patient experienced recurrent hemorrhages 4 months after the first ictus. This study describes treatment for moyamoya disease accompanied with distal choroidal artery aneurysms. Our experience suggests that cerebral revascularization combined with obliteration of the complicated distal aneurysm in the same session is a possible treatment.

## Background

Moyamoya disease is a chronic cerebrovascular disorder characterized by idiopathic stenosis or occlusion of the bilateral terminal internal carotid arteries. It is accompanied by the gradual development of a characteristic cerebrovascular collateral network, which resembles a “puff of smoke” on an angiogram [1,2]. The vascular changes observed in moyamoya disease can affect both adults and children, but their clinical features often differ. Most pediatric patients suffer primarily from transient ischemic strokes or cerebral infarction, whereas approximately half of the adult patients experience intracerebral or intraventricular hemorrhages, due to the rupture of cerebral aneurysms arising from moyamoya vessels [3,4].

Moyamoya disease associated with distal choroidal artery aneurysms is rarely encountered. When it occurs, the prognosis is very poor [3,5-7]. Unlike the common berry aneurysms arising from the circle of Willis, distal peripheral artery aneurysms associated with moyamoya disease are difficult to localize intraoperatively, and their direct surgical treatment is complicated by the risk of damaging the surrounding fine collateral network while exposing the distal peripheral artery aneurysm.

There is no unified treatment for patients with distal choroidal artery aneurysms associated with moyamoya disease. Some experts suggest that endovascular intervention is a suitable strategy for the management of distal choroidal artery aneurysms associated with moyamoya disease [8-10]. However, it has been confirmed that some aneurysms disappear spontaneously shortly after performing surgical cerebral revascularization because of the reduction of hemodynamic stress [11-13].

Three cases of distal choroidal artery aneurysms associated with moyamoya disease were encountered in our study and are described in this report. One case received conservative treatment. In the other two cases, superficial temporal artery (STA)-middle cerebral artery (MCA) anastomosis combined with encephalo-duro-myo-synangiosis (EDMS) and clipping or excision of the aneurysm were performed in the same operation, which has rarely been reported. In order to explore the feasible management of distal choroidal artery aneurysms in patients with moyamoya disease, we performed a literature review on this uncommon but challenging condition [10,12,14,15].

## Case presentation

### Case 1

**Figure 1:**
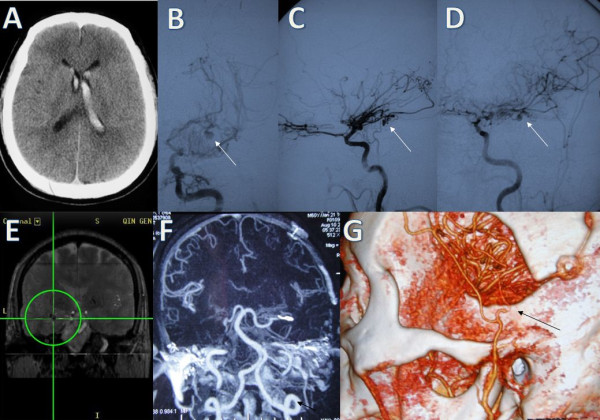
**Case 1. (A)** Computed tomography (CT) scan showing an intraventricular hemorrhage originating from the left subependymal area. **(B)**, **(C)**, **(D)** Left internal carotid angiograms revealing severe stenosis of the carotid fork accompanied with moyamoya vessels. There is an aneurysm (arrows) originating from the anterior choroidal artery **(B** frontal view, **C** lateral view, **D** oblique view**)**. **(E)** Surgical planning with neuronavigation demonstrating the surgical trajectory to the aneurysm. **(F)**, **(G)** CT scans 34 months after surgery showing the disappearance of the aneurysm and the patency of the bridge vessels (arrows).

A 51-year-old man was hospitalized for the sudden onset of a severe headache. Neurological examination revealed conjugate eye deviation to the right and a right flaccid hemiplegia. Computed tomography (CT) showed there was an intraventricular hemorrhage (IVH) originating from the left subependymal area (Figure 1A). Digital subtraction angiography (DSA) revealed severe stenosis of both supraclinoid internal cerebral arteries with moyamoya vessels. A 4-mm aneurysm was present at the left anterior choroidal artery (Figure 1B,C,D). An external ventricular drain was placed and the patient experienced progressive neurological improvement. A STA-MCA bypass combined with EDMS was performed, and the aneurysm was reached under the assistance of intraoperative neuronavigation (Figure 1E) and treated by direct neck clipping. The postoperative course was uneventful. A 34-month follow-up CT scan after surgery revealed a patent bypass with adequate perfusion through both the direct and indirect anastomoses (Figure 1F,G). The aneurysm was no longer visible. No recurrent hemorrhages occurred after surgery and his score on the Glasgow Outcome Scale (GOS) at follow-up was 5.

### Case 2

**Figure 2:**
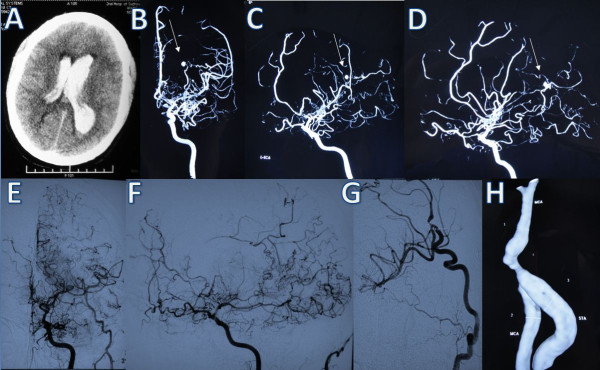
**Case 2. (A)** Computed tomography scan showing diffuse intraventricular hemorrhage. **(B)**, **(C)**, **(D)** Right carotid artery angiograms obtained after the hemorrhage showing marked stenosis of the proximal segment of the middle cerebral artery with a diffuse network of collateral fine vessels. There is an aneurysm (arrows) arising from the left posterior choroidal artery **(B** frontal view, **C** oblique view, **D** lateral view**)**. **(E)**, **(F)**, **(G)**, **(H)** DSA image at postoperative follow-up showing no residual aneurysm and an enlarged STA.

A 38-year-old man was admitted with a severe headache accompanied by vomiting. Neurological examination evidenced drowsiness and neck stiffness. CT revealed diffuse IVH (Figure 2A). The internal carotid artery angiograms showed bilateral severe focal stenosis of the proximal segment of the middle cerebral artery with a diffuse network of collateral fine vessels. A globular aneurysm arising from the left posterior choroidal artery was also visible (Figure 2B,C,D). The patient had a progressive recovery with external ventricular drainage. Two months later, he underwent a craniotomy, and the aneurysm was exposed through direct access to the posterior portion of the lateral ventricle and it was excised during surgery. During the same operation, a STA-MCA bypass combined with EDMS was performed. The patient had an uneventful postoperative course without fixed deficit, and follow-up DSA performed at 21 months after surgery showed no residual aneurysm; an enlarged STA suggested good distal function of the bypass (Figure 2E,F,G,H). No hemorrhage occurred after surgery and his GOS score at follow-up was 5.

### Case 3

**Figure 3:**
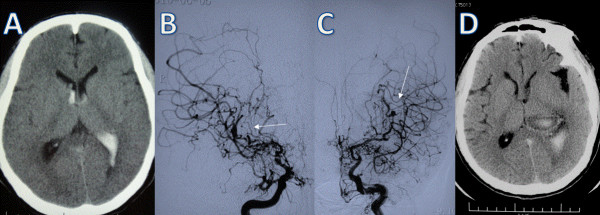
**Case 3. (A)** Computed tomography scan showing intraventricular hemorrhage involving the left lateral ventricle. **(B)**, **(C)** DSA demonstrating severe occlusive changes of both intracranial internal carotid arteries. There are two aneurysms (arrows) arising from the distal portion of the bilateral middle cerebral artery. **(D)** Computed tomography scan showing a recurrent intraventricular hemorrhage.

A 42-year-old man suffered from sudden onset of a severe headache followed by transient loss of consciousness. On admission, no neurological signs and symptoms were evident except for the headache. CT showed IVH with primary involvement of the left lateral ventricle (Figure 3A). DSA demonstrated severe occlusive changes of both intracranial internal carotid arteries, which was suggestive of moyamoya disease. Additionally, two aneurysms were found. One was in the right lateral ventricle located at the distal portion of the right anterior choroidal artery, while the other was supplied by the left anterior choroidal artery (Figure 3B,C). The patient was treated conservatively, and his neurological symptoms gradually improved. After consulting with the patient and his family about the potential treatment options, their efficacy and risks, they declined the surgery and opted for conservative management. However, 4 months later the patient experienced a recurrent IVH (Figure 3D). Surgery was recommended again, but his family declined and the patient was managed conservatively.

### Discussion

In this report, we describe three patients with IVH secondary to distal aneurysms associated with moyamoya disease. In the literature there are five other cases of ruptured distal aneurysm accompanying moyamoya disease. The clinical data for these five cases are summarized in Table 1.

**Table 1:** Clinical data for the reported cases of ruptured distal aneurysms accompanying moyamoya disease

**Authors/year**	**Age/sex**	**Presentation**	**Treatment**	**Pathology**	**Outcome**
Kuroda *et al*. [12]/2001	60 yrs/F	IVH	Revascularization	Not performed	Good
Ali *et al*. [14]/2004	26 yrs/M	ICH & IVH	Conventional clipping	True aneurysm	Good
Nishio *et al*. [10]/2004	47 yrs/F	SAH	Embolization	Not performed	NR
Gandhi *et al*. [15]/2008	59 yrs/M	SAH	Conventional clipping	Not performed	NR
Lévêque *et al*. [16]/2011	50 yrs/F	IVH	Endoscopy-assisted surgery	Not performed	NR

*F*, Female; *ICH*, Intracerebral hemorrhage; *IVH*, Intraventricular hemorrhage; *M*, Male; *NR*, Not reported; *SAH*, Subarachnoid hemorrhage.

Kuroda *et al*. [12] described the case of a 60-year-old female with moyamoya and IVH. Cerebral angiography revealed that the bleeding resulted from the rupture of peripheral artery aneurysms arising from dilated collateral vessels. She successfully underwent STA-MCA anastomosis combined with EDMS and recovered well. Ali *et al*. [14] described the case of a 26-year-old male patient with an intracerebral hemorrhage (ICH) and IVH. Two aneurysms were suggested: a 2-mm aneurysm supplied by prominent thalamic perforators on the left, and a 6- to 7-mm aneurysm located at the distal portion of the hypertrophied right lateral posterior choroidal artery, which was shown to be a true saccular aneurysm. Frameless stereotactically guided trapping and microsurgical excision of the intraventricular aneurysm were performed. Nishio *et al*. [10] described the case of a 47-year-old female with moyamoya disease associated with a ruptured aneurysm located at the P1 segment of the left posterior cerebral artery. CT found a subarachnoid hemorrhage (SAH). Endovascular embolization was performed using a Guglielmi detachable coil, and the aneurysm was completely occluded with preservation of the parent artery. Gandhi *et al*. [15] described the case of a 59-year-old male with a ruptured lenticulostriate artery aneurysm and SAH. The patient underwent a pterional craniotomy and clipping of the aneurysm. The outcome was not reported. Lévêque *et al*. [16] treated a 50-year-old female presenting with IVH twice as a result of moyamoya disease. An aneurysm located on the left distal anterior choroidal artery was observed using cerebral angiography. The aneurysm was accurately reached endoscopically and successfully resected from the parent artery.

Of the five patients, two were males and three were females. They ranged in age from 26 to 60 years. The types of onset included IVH in two cases, IVH with ICH in one case, and SAH caused by rupture of another type of aneurysm in two cases. CT or DSA were performed to provide an accurate diagnosis in all cases. All aneurysms were ruptured. One patient received endovascular embolization, two cases were subjected to conventional aneurysm clipping, one case experienced endoscopy-assisted surgery and one underwent revascularization. The histopathology of one aneurysm was consistent with a true saccular aneurysm. Two of these patients had a good prognosis, while the prognoses for the others were not reported.

Given the paucity of cases reported, there is no consensus about their optimal management. The clinical characteristics of the three patients with hemorrhagic moyamoya disease in our study are summarized in Table 2. In the first two patients, we treated the aneurysm (by neck clipping in one case and parent vessel sacrifice in the other) combined with direct and indirect revascularization in the same sitting. The rationale for performing revascularization in these cases is based on the hypothesis that the aneurysms are induced by increased hemodynamic stress to the perforating vessels, which participate in the collateral blood flow in moyamoya disease. Providing additional sources of collateral blood flow through direct and indirect anastomoses in these patients should in theory decrease the hemodynamic stress on the delicate collateral network of vessels and, thus, hopefully decrease the risk of *de novo* aneurysm formation. Unfortunately, the deep aneurysms associated with moyamoya disease are difficult to localize. However, through the aid of CT angiography based on neuronavigation, it is feasible to localize an aneurysm accurately and to steer away from eloquent areas, with the additional benefit of a smaller cortical incision and reduced risk of damaging fine collaterals by planning the surgical trajectory. These considerations are extremely important as the brain in this condition has a very poor tolerance to ischemia and surgical manipulation. As the procedures in our two patients were delayed, aneurysm localization was facilitated by the observation of a dense hemosiderin stain around the aneurysm. Further analysis confirmed that this indeed was the site of rupture. Once the aneurysm is exposed, direct clipping or parent artery sacrifice and trapping are then used to obliterate the aneurysm definitively, since the most distal portion of the choroidal vessels participate almost exclusively in the supply of the choroid plexus, and it is safe to sacrifice them.

**Table 2:** Summary of clinical characteristics of three patients with hemorrhagic moyamoya disease accompanied by intracranial aneurysms

	**Patient 1**	**Patient 2**	**Patient 3**
Age (years)/sex	51/M	38/M	42/M
Diagnosis	IVH	IVH	IVH
Affected artery	Anterior choroidal	Posterior choroidal	Bi-anterior choroidal
Suzuki’s vessel grades	III	III	III
Preoperative GCS	15	15	15
Operation	STA-MCA + EDMS and aneurysm neck clipping	STA-MCA + EDMS and aneurysmectomy	None
Postoperative aneurysm	Disappeared	Disappeared	Remains
STA-MCA	++	++	--
EDMS	++	++	--
Follow-up period (months)	34	21	4
Rebleeding	None	None	Yes
GOS	5	5	5

*EDMS*, Encephalo-duro-myo-synangiosis; *F*, Female; *GCS*, Glasgow Coma Score; *GOS*, Glasgow Outcome Scale; *IVH*, Intraventricular hemorrhage; *M*, Male; *STA-MCA*, Superficial temporal artery-middle cerebral artery anastomosis; ++, moderate development.

A hemorrhage is the major catastrophic issue in the natural course of hemorrhagic moyamoya disease [2,10]. Clinically, the treatment for this type of moyamoya disease is always based on the cause and location of the hemorrhage, which occurs primarily for three reasons: rupture of accompanying aneurysms, rupture of moyamoya vessels or rupture of choroidal arteries [3,17,18]. However, as with all causes of hemorrhage, prevention of the rebleeding in moyamoya disease is important in treatment. Direct STA-MCA anastomosis combined with EDMS for treating hemorrhagic moyamoya disease are worthy of more attention. Multi-center retrospective questionnaires [19,20] have revealed that the annual rate of rebleeding was noticeably lower for patients who experienced an STA-MCA bypass combined with EDMS, compared with those who were treated conservatively. More importantly, our results are consistent with these studies. On the other hand, the presence of a distal aneurysm accompanying moyamoya disease, to a large extent, has a bearing on the prognosis of hemorrhagic moyamoya disease. Kawaguchi *et al*. [3] pointed out that this type of distal artery aneurysm is known to rupture more than twice. They enumerated 44 cases with 58 aneurysms, which were separately identified within moyamoya vessels, the distal posterior cerebral artery, the anterior and posterior choroidal arteries and so on. Scores on the GOS for 34 patients (77.2%) were 1, 2 or 3 after the onset of hemorrhage [3]. Moreover, another report [21] showed that aneurysms accompanying moyamoya disease have a tendency for early rebleeding. The third patient presented in this report also supported these findings, since he suffered rebleeding four months after his first hemorrhage, and the cause of the bleeding was believed to be an aneurismal rupture. Therefore, our data also indicate that these patients have a risk of rebleeding and a poor prognosis, and cerebral revascularization with simultaneous obliteration of the aneurysm could be an efficient way to prevent rebleeding. Some authors [12] advocate that a distal aneurysm should be indirectly obliterated via revascularization. One possible mechanism is that surgical revascularization decreases the hemodynamic stress, reducing the blood flow to normal and indirectly ameliorating the natural history of precedential ruptured distal aneurysms among patients with moyamoya disease. However, another report [22] indicated that the mechanism for the disappearance of distal aneurysms is spontaneous thrombosis and that the hemodynamic stress on collateral vessels remained too high.

According to the outcome of these peripheral artery aneurysms associated with moyamoya disease, we hypothesize that the two mechanisms coexist. Bypass surgery can improve the impaired intracranial circulation and partly relieve hemodynamic stress, but the problem is not completely solved. Although aneurysms do not develop within the angiography, the parent arteries are often consistent in caliber [7,23]. This is direct evidence in support of our theory that there is still hemodynamic stress. In the two patients we reported, cerebral revascularization was used to improve the abnormal hemodynamic stress and the obliteration of the aneurysms prevented recurrent hemorrhages in the short term. An endovascular embolization [24-26] with coils or liquid materials is also considered, but it often carries a risk of occlusion of the parent artery because of the highly tortuous route to the aneurysm and the small caliber of the vessel. So the possibility of endovascular treatment is a less attractive option.

We acknowledge two limitations to this study. First, while the results of the follow-ups have been satisfactory, a longer-term follow-up will be important role for the proper evaluation of this modality of treatment. Second, this study was not prospective and further prospective cases and a control study will be conducted in the future.

## Conclusions

A patient with hemorrhagic moyamoya disease accompanied with distal aneurysm has a tendency for rebleeding. Our experience in these three cases indicated that revascularization combined with direct obliteration of the aneurysms is an efficient way to prevent rebleeding.

## Consent

Written informed consent was obtained from the patients for publication of this case report and all accompanying images. A copy of the written consent is available for review by the Editor-in-Chief of this journal.

## Abbreviations

CT: Computed tomography; DSA: Digital subtractive angiography; EDMS: Encephalo-duro-myo-synangiosis; GCS: Glasgow Coma Score; GOS: Glasgow Outcome Scale; ICH: Intracerebral hemorrhage; IVH: Intraventricular hemorrhage; MCA: Middle cerebral artery; SAH: Subarachnoid hemorrhage; STA: Superficial temporal artery.

## Competing interests

The authors declare that they have no competing interests.

## Authors’ contributions

KH and WZ conceived and designed the study. LC provided the study material. YM collected and assembled the data. YM wrote the manuscript. All authors read and approved the final manuscript.
